# Recent Clinical and Molecular Advances in the Management of Thymic Carcinoids: A Comprehensive Review

**DOI:** 10.3390/cancers17121975

**Published:** 2025-06-13

**Authors:** Aleksandra Piórek, Adam Płużański, Dariusz M. Kowalski, Maciej Krzakowski

**Affiliations:** Department of Lung Cancer and Thoracic Tumors, Maria Sklodowska-Curie National Research Institute of Oncology, 02-781 Warsaw, Poland; adam.pluzanski@nio.gov.pl (A.P.); dariusz.kowalski@nio.gov.pl (D.M.K.); maciej.krzakowski@nio.gov.pl (M.K.)

**Keywords:** thymic carcinoid, thymic neuroendocrine tumor, mediastinal tumor, targeted therapy, immunotherapy

## Abstract

**Background:** Thymic carcinoids are rare neuroendocrine tumors arising in the anterior mediastinum, often diagnosed at an advanced stage due to nonspecific clinical manifestations. Their management remains challenging because of the paucity of data, rarity of occurrence, and aggressive biological behavior compared to other well-differentiated neuroendocrine neoplasms. **Methods:** We conducted a comprehensive review of the current literature focusing on the classification, clinical presentation, diagnostics, treatment options, prognostic factors, and emerging experimental therapies for thymic carcinoids. Emphasis was placed on integrating recent molecular and therapeutic advances into clinical practice. **Results:** Surgical resection remains the cornerstone of treatment for localized disease, while systemic therapies such as everolimus, somatostatin analogs, platinum-based chemotherapy, and peptide receptor radionuclide therapy (PRRT) are options for advanced cases. Novel diagnostic modalities, including NETest, 64Cu-DOTATATE PET, and 18F-FDOPA PET, offer promise in early detection and disease monitoring. Molecular insights, particularly involving MEN1, ATRX, and DAXX mutations, pave the way for individualized targeted therapies. Immunotherapy and radioimmunotherapy represent emerging, albeit still experimental, approaches. Prognosis largely depends on tumor stage, differentiation, resectability, and functional activity, with a high recurrence rate necessitating prolonged surveillance. **Conclusions:** Thymic carcinoids pose significant diagnostic and therapeutic challenges. Advances in molecular profiling, novel imaging techniques, and systemic therapies offer hope for improved outcomes. Given the disease rarity, continued collaboration through registries and multicenter studies is essential to refine evidence-based management strategies.

## Simple Summary

Thymic carcinoids are extremely rare tumors that are often difficult to detect in their early stages. Because of their low incidence, clinical experience with their management is limited, and large-scale studies are scarce. This review brings together the most recent information on how to recognize, diagnose, and treat thymic carcinoids. It explains how new tests, imaging techniques, and targeted therapies are changing the way these tumors are managed. We also highlight ongoing research on experimental treatments, such as immunotherapy. By combining clinical observations with emerging scientific knowledge, this article aims to support improved care for patients with this rare disease.

## 1. Introduction

Thymic neuroendocrine tumors (TNETs) are a rare and heterogeneous group of neoplasms that arise from neuroendocrine cells of the thymus. They represent less than 5% of all thymic malignancies and approximately 0.4% of all neuroendocrine tumors. According to SEER data, the incidence of TNETs is extremely low, estimated at 0.02–0.04 per 100,000 persons per year, and they account for only 2% of all mediastinal tumors [1,2]. Despite their rarity, TNETs are notable for their aggressive clinical behavior, high potential for metastasis, and diagnostic complexity.

Historically, TNETs were grouped with pulmonary carcinoids due to overlapping histological features. However, current evidence indicates distinct differences in their biology, clinical course, and genetic background. The WHO classification (2015) distinguishes four categories of thymic neuroendocrine neoplasms based on differentiation and mitotic activity: typical carcinoid (TC), atypical carcinoid (AC), large cell neuroendocrine carcinoma (LCNEC), and small cell carcinoma (SCC) [3]. While TC and AC are considered well-differentiated neoplasms (traditionally referred to as “thymic carcinoids”), LCNEC and SCC represent poorly differentiated high-grade tumors [2,4].

Compared with their pulmonary counterparts, TNETs tend to present at a more advanced stage, have lower resection rates, and are associated with poorer overall survival outcomes. For instance, 5-year survival rates range from 50 to 100% for TC, 30 to 60% for AC, and below 30% for high-grade NECs [2]. Up to 50% of cases may exhibit functional hormonal activity, with ectopic ACTH secretion being the most common syndrome, leading to paraneoplastic Cushing’s syndrome. TNETs, particularly ACs, are also significantly associated with multiple endocrine neoplasia type 1 (MEN-1) in approximately 25% of cases, though this varies across studies [2].

Due to their rarity, the optimal approach to diagnosis, staging, and treatment remains a challenge, and existing guidelines are based primarily on limited retrospective data and extrapolation from pulmonary carcinoid management. Recent advances in imaging, pathology, and molecular characterization are beginning to refine the understanding of TNETs and their subtypes. This article provides an overview of thymic neuroendocrine tumors, with particular emphasis on thymic carcinoids (TC and AC), which constitute the primary focus of this review. Key topics include classification, epidemiology, clinical presentation, diagnostics, treatment modalities, and prognostic markers. Special emphasis is placed on recent molecular advances, systemic therapeutic strategies, and the evolving landscape of experimental approaches. By integrating these insights, we aim to support evidence-based management of this rare and challenging disease.

## 2. Definition and Classification

Thymic carcinoids are rare NETs most commonly located in the thymus (anterior mediastinum), classified within the broader group of thymic neuroendocrine tumors (TNETs). Thymic carcinoids account for only a small percentage of all NETs and all primary tumors of the thymus.

According to the World Health Organization (WHO) classification (2015, confirmed in 2021) [5,6], pulmonary and thymic carcinoids are categorized into four histological types:

• Typical carcinoid (TC; well-differentiated, low-grade)—a well-differentiated neoplasm characterized by low histological aggressiveness (usually <2 mitoses per 2 mm^2^ and absence of necrosis);
• Atypical carcinoid (AC; intermediate-grade)—a tumor of intermediate malignancy, displaying features of histological atypia, such as foci of necrosis and/or increased mitotic activity (2–10 mitoses per 2 mm^2^);
• Large-cell neuroendocrine carcinoma (LCNEC; high-grade)—a poorly differentiated neoplasm composed of large cells, with a high mitotic rate (>10 mitoses per 2 mm^2^) and areas of necrosis, but lacking the morphological features of small-cell carcinoma;
• Small-cell carcinoma (SCC; high-grade)—a highly aggressive, poorly differentiated neuroendocrine tumor with characteristic small-cell morphology.

Typical and atypical carcinoids are considered well-differentiated NETs, whereas LCNEC and small-cell carcinoma are classified as poorly differentiated neuroendocrine carcinomas. It is noteworthy that, within the thymus, atypical carcinoids are significantly more frequent than typical carcinoids, with an approximate ratio of 2:1 [6,7].

A comparison between typical and atypical carcinoids is presented in Table 1.

## 3. Epidemiology

Thymic carcinoids are among the rarest forms of NETs. The estimated annual incidence is approximately 0.02–0.2 cases per 100,000 individuals [5]. They account for less than 0.5% of all NETs and approximately 2–5% of all malignant tumors of the thymus [1,6,8]. In contrast, bronchopulmonary carcinoids are significantly more common, representing about 20–25% of all NETs [5].

Thymic carcinoids predominantly occur in middle-aged individuals, with a median age at diagnosis of approximately 54 years [1,8]. There is a marked male predominance, with a reported male-to-female ratio ranging from 3:1 to 5:1 in different series [1].

The etiology of sporadic thymic carcinoids remains unknown, and no clear association with environmental factors or carcinogen exposure has been established. However, genetic predisposition plays a significant role. TNETs are part of the broad clinical spectrum of multiple endocrine neoplasia type 1 (MEN1), a hereditary tumor syndrome caused by germline mutations in the MEN1 gene. While MEN1-associated tumors most commonly include parathyroid, pancreatic, and pituitary neoplasms, approximately 25% of all TNETs have been reported to occur in the setting of MEN1 [2,9,10,11]. Conversely, in large MEN1 patient cohorts, only 3.1–8.2% develop thymic NETs, reflecting their relatively low overall prevalence in this population [9,11,12,13]. Despite their rarity, TNETs represent a significant source of morbidity and mortality in MEN1 patients, due to their greater malignant potential, higher risk of local invasion, and early metastasis. For this reason, biannual thoracic imaging with CT or MRI is recommended for MEN1 patients to enable early detection. Some authors also propose prophylactic thymectomy, particularly when performed concurrently with parathyroidectomy, to reduce the future risk of developing thymic NETs [14]. In the specific context of ACTH-producing TNETs associated with ectopic Cushing’s syndrome (ECS), MEN1 mutations were reported in only a small proportion of cases (around 3%), although this is likely an underestimate due to incomplete genetic testing in many published reports. Routine MEN1 screening in all patients with ECS-related TNETs does not appear necessary at this time, given the low frequency of MEN1 mutations and the typically overt clinical presentation of ECS [9,15,16].

## 4. Clinical Presentation

The clinical presentation of mediastinal carcinoids is often mild or nonspecific for an extended period. More than 30% of patients are asymptomatic at the time of diagnosis, with tumors discovered incidentally during imaging performed for unrelated reasons [17]. In symptomatic individuals, clinical manifestations result from two primary mechanisms: the mass effect of the tumor within the mediastinum (due to compression or invasion of adjacent structures), and hormone secretion leading to paraneoplastic syndromes [8,18].

Typical symptoms related to a mass in the anterior mediastinum include persistent cough, dyspnea, a sensation of chest tightness or pain, and occasionally hoarseness, which may occur because of recurrent laryngeal nerve involvement. Superior vena cava syndrome (SVCS), characterized by facial swelling, cyanosis, and jugular vein distension, is observed in approximately 20% of patients and results from vascular compression or thrombosis [19]. At the time of diagnosis, around half of patients already have invasion of adjacent structures such as the pericardium, lungs, or great vessels, and distant metastases are present in 20–30% of cases [6,18,20].

In contrast to carcinoids of the gastrointestinal tract or lung, thymic carcinoids rarely cause the classical carcinoid syndrome (flushing, diarrhea, and bronchospasm) [2,6,21]. TNETs are one of the recognized sources of ectopic adrenocorticotropic hormone (ACTH) production, leading to ECS—a rare but clinically significant paraneoplastic manifestation. ECS occurs in approximately 20–40% of patients with TNETs, significantly more often than in NETs of other locations [6,9,22,23,24,25,26,27]. Histologically, ECS is most commonly associated with thymic AC. In a recent systematic review of 162 reported cases of ectopic Cushing’s syndrome caused by thymic neuroendocrine tumors, 46.7% were identified as atypical carcinoids, followed by typical carcinoids (30.4%) and high-grade neuroendocrine carcinomas, including large-cell and small-cell subtypes (21.7%) [9]. This distribution underscores the prominent role of intermediate-grade thymic NETs—especially AC—in the pathogenesis of ACTH-producing tumors. Notably, ACTH-secreting tumors are more likely to exhibit aggressive clinical behavior and are often diagnosed at an advanced stage [9]. Patients often present with rapid-onset and severe manifestations of hypercortisolism, including refractory hypertension, hypokalemia, new-onset diabetes, muscle weakness, and metabolic alkalosis [22]. These symptoms may dominate the clinical picture and precede the diagnosis of the underlying tumor, sometimes by several months. Unlike pituitary Cushing’s disease, the clinical course in ECS is typically more aggressive, with frequent complications such as opportunistic infections, cardiovascular events, and thromboembolic phenomena. Diagnosis is based on a combination of biochemical markers (elevated ACTH and cortisol, lack of suppression in dexamethasone tests), and imaging—most commonly contrast-enhanced CT or MRI of the mediastinum. Thymic tumors associated with ECS tend to be larger, have a higher rate of invasion and metastasis, and are associated with worse prognosis compared to non-secreting counterparts [9]. The first-line treatment of ACTH-producing TNETs is surgical resection, which leads to rapid reversal of hypercortisolism and improvement in clinical symptoms [9]. However, many patients present with advanced disease, rendering complete resection difficult. In such cases, cortisol-lowering medical therapy (e.g., metyrapone, ketoconazole, osilodrostat) and bilateral adrenalectomy are considered as temporizing or palliative interventions [9]. Given its high morbidity and direct impact on survival, early recognition of ECS in patients with suspected or known TNETs is critical. Multidisciplinary management involving endocrinologists, oncologists, and thoracic surgeons is essential for optimal outcomes [9].

Another relatively frequent paraneoplastic manifestation is hypercalcemia, observed in about 20–30% of patients, attributed to ectopic secretion of parathyroid hormone-related peptide (PTHrP) [6]. Isolated case reports have also described ectopic production of growth hormone-releasing hormone (GHRH), leading to acromegaly, and antidiuretic hormone (ADH), resulting in the syndrome of inappropriate antidiuretic hormone secretion (SIADH, or Schwartz–Bartter syndrome); however, these occurrences are exceedingly rare [6].

## 5. Diagnostics

The diagnosis of mediastinal carcinoids is based on a combination of clinical evaluation, imaging studies, and histopathological confirmation. Given the nonspecific nature of symptoms, abnormal imaging of the chest is often the first indicator of disease.

The primary imaging modality is contrast-enhanced chest CT, which allows for visualization of the mediastinal mass, including its size, characteristics, and relationship to surrounding structures [8]. Thymic carcinoids typically present as large, heterogeneous anterior mediastinal masses with irregular borders, often exhibiting calcifications, areas of necrosis, or hemorrhage [28]. These tumors frequently reach considerable size before clinical detection. Magnetic resonance imaging (MRI) may be particularly useful in selected cases, especially for assessing invasion of the heart, aorta, or pulmonary trunk. On T2-weighted sequences, thymic carcinoids often appear heterogeneous, and post-contrast images commonly reveal intense enhancement with intralesional septations, particularly in atypical variants [28].

Functional imaging includes positron emission tomography (PET) with somatostatin receptor analogs labeled with Gallium-68 (e.g., 68Ga-DOTATATE or DOTATOC) or traditional somatostatin receptor scintigraphy (Octreoscan), which detect receptor expression by NET cells [29]. Similar to other well-differentiated NETs, thymic carcinoids typically exhibit strong somatostatin receptor expression. These scans aid in differentiating NETs from other anterior mediastinal tumors and, importantly, a positive result supports eligibility for peptide receptor radionuclide therapy [30]. Standard 18F-FDG PET may also be used to assess distant metastases, although well-differentiated NETs generally show low FDG avidity. In contrast, more aggressive tumors, such as LCNEC and small-cell carcinoma, typically demonstrate high FDG uptake [29].

Definitive diagnosis relies on histopathological examination, including immunohistochemistry. If the tumor is operable, complete surgical excision followed by pathological assessment is the preferred approach. In cases of large, inoperable tumors or diagnostic uncertainty, core-needle biopsy under CT guidance is commonly performed. Histologically, thymic carcinoids exhibit a characteristic neuroendocrine morphology, typically composed of nests or trabeculae of uniform cells with “salt-and-pepper” chromatin. Immunohistochemical staining is typically positive for neuroendocrine markers such as chromogranin A and synaptophysin [6], and frequently also CD56 [6]. In contrast, epithelial markers such as cytokeratin 5/6—typically seen in thymomas—are usually negative, helping to distinguish these tumors [6]. Thyroid transcription factor-1 (TTF-1) immunostaining may assist in determining the tumor origin; for example, a pulmonary NET metastatic to the thymus would typically be TTF-1-positive, whereas a primary thymic carcinoid is usually TTF-1-negative [6].

Tumor grading is primarily based on World Health Organization (WHO) mitotic and morphological criteria. Although Ki-67 is not formally included in WHO grading for thymic carcinoids, it is frequently assessed. A Ki-67 index <3% is consistent with low-grade tumors (G1), while a value >20% indicates high-grade tumors (G3). However, exceptions may occur—for instance, well-differentiated tumors may present with elevated Ki-67 values >20% [7].

In suspected cases of ectopic ACTH secretion, evaluation includes measurement of morning serum cortisol and ACTH, as well as dexamethasone suppression testing (elevated, non-suppressible cortisol levels support the diagnosis of Cushing’s syndrome) [31]. For suspected ectopic GHRH production, serum IGF-1 and GHRH levels are assessed; in the case of PTHrP secretion, serum calcium, PTH, and PTHrP levels are measured. These biomarkers are useful not only for diagnosis of paraneoplastic syndromes but also for monitoring disease activity during treatment [31].

Tumor staging is currently performed using the TNM classification for thymic malignancies, as defined by the 8th edition of the UICC/AJCC system [5]. Alternatively, the historical Masaoka-Koga classification—originally developed for thymomas—is occasionally used [6]. At the time of diagnosis, most patients present with stage III or IV disease, reflecting local invasion or distant metastases [6]. Unfortunately, a dedicated TNM staging system specific to thymic NETs has not yet been established.

The European Neuroendocrine Tumor Society (ENETS) has proposed a general NET classification system that includes both histological grade (based on Ki-67 index: G1 <3%, G2 3–20%, G3 >20%) and TNM-like staging [5]. For thymic carcinoids, ENETS recommends assessing tumor grade in conjunction with WHO histopathological criteria, even though formal diagnosis is based primarily on morphology.

## 6. Molecular Profiling and Emerging Genomic Insights

Molecular data on TNETs, particularly thymic carcinoids (typical and atypical), remain sparse due to their rarity. Nonetheless, several studies employing next-generation sequencing (NGS) approaches have revealed key genomic alterations [2,32].

In a multicenter study involving 73 TNETs, Ströbel et al. demonstrated a clear correlation between chromosomal complexity and histological grade. The average number of chromosomal aberrations per tumor was 0.8 in typical carcinoids (TC), 1.1 in atypical carcinoids (AC), and 4.7 in high-grade neuroendocrine carcinomas (LCNEC and SCC). Amplification of the 8q24 region, which harbors the MYC oncogene, was the most frequent alteration across all subtypes [33].

Similarly, Rieker et al. identified chromosomal imbalances in 8 out of 10 sporadic TNETs. Common gains included Xp, 7p, 7q, 11q, 12q, and 20q, while recurrent losses occurred at 6q, 6p, 4q, 3p, 10q, 11q, and 13q. These findings suggest partial genomic overlap with advanced thymomas [15].

In thymic carcinoids, deletions affecting the MEN1 gene locus on 11q have been reported, similar to pulmonary carcinoids. In lung carcinoids, biallelic somatic inactivation of MEN1 has been observed in up to 36% of cases, and analogous mechanisms may occur in thymic tumors [2,34]. However, MEN1-related TNETs show fewer 11q13 losses compared to pancreatic or parathyroid MEN1-associated neoplasms, indicating a distinct tumorigenic pathway [2].

Whole-exome sequencing in thymic NETs associated with ectopic ACTH secretion has identified non-recurrent somatic mutations in HRAS, PAK1, and MEN1, suggesting substantial genetic heterogeneity and emphasizing the need for broader molecular studies [2].

NGS analyses, although performed in small patient cohorts, have identified recurrent alterations in several genes [2,15,33,35,36]. The most frequently reported mutations include MEN1, TP53, HRAS, NF1, KIT, and ATRX. Mutations in the MEN1 gene are particularly relevant, not only in the context of inherited MEN-1 syndrome but also in sporadic cases of AC. These alterations suggest an overlap with pancreatic and pulmonary neuroendocrine tumors but also point to unique molecular features of thymic origin. [2].

Moreover, targeted next-generation sequencing (NGS) conducted by Sakane et al. in a cohort including six thymic neuroendocrine tumors revealed actionable genomic alterations. The most frequently mutated gene was TP53 (18.5%), followed by KIT (7.4%) and PDGFRA (5.6%). Genes involved in the p53 pathway were most commonly affected (20.4%), followed by the receptor tyrosine kinase (RTK)/RAS (18.5%) and PI3K (5.6%) pathways. Notably, mutations in EGFR were observed in a minority of cases (3.7%), and RTK pathway mutations were associated with worse overall survival, underscoring their prognostic significance. Nearly 19% of cases harbored potentially targetable mutations, highlighting the promise of precision oncology in this setting [32].

High-grade TNETs (LCNEC and SCC) are characterized by molecular profiles consistent with poorly differentiated phenotypes. These include frequent TP53 mutations, RB1 loss, and global chromosomal instability. Immunohistochemistry often confirms loss of the Rb protein and aberrant p53 overexpression, in line with these molecular findings [36].

At present, no definitive molecular classification has been established for TNETs, in contrast to the well-characterized molecular subgroups in lung or gastrointestinal neuroendocrine neoplasms. This represents a major obstacle for the development of biomarker-driven personalized therapies in this group.

The tumor microenvironment and immune landscape of TNETs are also being investigated. In one recent study, PD-L1 expression and the presence of tumor-infiltrating lymphocytes (TILs) were observed in a subset of AC and LCNEC cases, suggesting possible responsiveness to immune checkpoint blockade [37].

## 7. Treatment

Management of thymic carcinoids should be conducted in centers with expertise in NETs, ideally involving a multidisciplinary team comprising a thoracic surgeon, medical oncologist, endocrinologist, and radiation oncologist. Due to the rarity of this tumor type, no randomized clinical trials have been conducted, and current treatment strategies are based on observational data, case series, and clinical guidelines primarily derived from pulmonary carcinoids. Table 2 provides an overview of therapeutic strategies for thymic carcinoids according to disease stage.

### 7.1. Surgical Treatment

Radical surgical resection is the primary and most effective treatment modality for patients with localized disease [22]. Complete resection of the tumor, including total thymectomy with en bloc removal of surrounding mediastinal fat and mediastinal lymph nodes, is recommended. This approach is analogous to the surgical management of other thymic tumors, aiming for complete excision of the anterior mediastinal compartment [5].

Unfortunately, in a substantial proportion of patients (approximately 50–70%), the tumor is already locally advanced or bulky at the time of diagnosis, rendering complete (R0) resection unfeasible [18]. Neoadjuvant chemotherapy or chemoradiotherapy has been described in selected cases to downstage large thymic carcinoids, but its efficacy remains unclear due to a lack of robust data [18,31]. If the tumor is initially unresectable and the patient’s general condition permits, induction therapy may be considered [31].

In patients with limited disease, surgical resection should be reconsidered even in the event of local recurrence, provided the lesion is technically resectable, as this may offer prolonged survival. Solitary distant metastases—such as those in the lungs or liver—may also be amenable to surgical removal in selected cases [5,31].

### 7.2. Radiotherapy

Radiotherapy (RT) plays a limited role in general but sometimes it may be used in the treatment of thymic carcinoids. Adjuvant RT following incomplete resection (R1/R2) is occasionally employed in clinical practice, although its impact on long-term survival is uncertain [22]. Such decisions should be made on a case-by-case basis within a multidisciplinary tumor board setting [5].

Palliative radiotherapy is effective in relieving symptoms caused by locally advanced disease, including SVCS, chest wall pain due to invasion, or bone metastases.

### 7.3. Systemic Therapy

According to the 2021 ESMO guidelines, several systemic treatment options may be considered for advanced thymic carcinoids. These include temozolomide (± capecitabine), platinum-based regimens (preferably with oxaliplatin), targeted therapy with everolimus, peptide receptor radionuclide therapy (PRRT), and interferon-α for carcinoid syndrome resistant to somatostatin analogs (SSA) [5].

#### 7.3.1. Chemotherapy

There is no standardized chemotherapy protocol specifically dedicated to thymic carcinoids; regimens are extrapolated from treatments used for pulmonary and gastrointestinal NETs. In poorly differentiated tumors (e.g., LCNEC, small-cell carcinoma), platinum–etoposide-based regimens are considered standard, similar to those used in small-cell lung cancer [18]. In typical or atypical carcinoids (well-differentiated), chemotherapy is less effective due to the indolent nature of these tumors and their lower chemosensitivity. In clinical practice, regimens established for other low- and intermediate-grade NETs (G1/G2)—such as streptozotocin with 5-fluorouracil or temozolomide with capecitabine—are sometimes utilized for advanced thymic carcinoids. However, available data on their efficacy are limited and inconsistent. Chemotherapy is considered when surgical options, somatostatin analogs, and other modalities have failed or are not feasible.

#### 7.3.2. Somatostatin Analogs

Most well-differentiated thymic carcinoids express somatostatin receptors, enabling the use of somatostatin analogs (SSA) for both diagnostic and therapeutic purposes. SSA agents such as octreotide and lanreotide are well-established for symptom control in gastrointestinal NETs. Although large clinical studies are lacking in thymic carcinoids, SSA therapy is considered in patients with advanced disease showing somatostatin receptor expression in imaging. In hormonally active cases (e.g., ectopic ACTH secretion), SSA may also help manage the associated endocrine symptoms [5]. Because of the rarity of these tumors, definitive evidence regarding survival benefit from SSA is lacking. Nonetheless, octreotide or lanreotide may be administered on an individualized basis, particularly when other treatment options are limited, given their favorable safety profile [31].

#### 7.3.3. Targeted Therapy

In studies involving advanced pulmonary NETs, everolimus has been shown to significantly prolong progression-free survival compared to placebo, as demonstrated in the RADIANT-4 trial [38]. Based on these results, clinical guidelines recommend everolimus as a second-line treatment option for patients with typical or atypical carcinoids of the lung or thymus who have experienced disease progression despite treatment with somatostatin analogs. Case reports have documented favorable outcomes with everolimus in thymic carcinoids, including prolonged disease stabilization lasting several months [7,39].

Other molecularly targeted therapies, such as angiogenesis inhibitors (e.g., sunitinib, pazopanib), may be considered in clinical trials [40].

#### 7.3.4. Peptide Receptor Radionuclide Therapy (PRRT)

Peptide receptor radionuclide therapy (PRRT) is an established systemic treatment for advanced NETs, involving intravenous administration of somatostatin analogs labeled with radioactive isotopes. Although large prospective trials in pulmonary and thymic carcinoids are lacking, retrospective analyses have suggested potential efficacy of PRRT in these subtypes [7].

Current clinical guidelines recommend PRRT as a third-line treatment for patients with metastatic pulmonary or thymic carcinoids who have progressed despite prior therapy with SSA and everolimus [5]. Eligibility for PRRT requires documented somatostatin receptor expression in all known tumor lesions and the absence of other suitable clinical trial options offering access to more advanced investigational therapies. PRRT is generally well tolerated, although patients require monitoring for renal function and bone marrow suppression.

#### 7.3.5. Immunotherapy

Immunotherapy—particularly immune checkpoint inhibitors targeting the PD-1/PD-L1 axis—has transformed the treatment landscape for several malignancies. However, its efficacy in neuroendocrine tumors remains limited. Well-differentiated NETs typically exhibit a very low tumor mutational burden (TMB), which may contribute to poor immunogenicity. In a phase II study, the use of immunotherapy monotherapy in well-differentiated NETs yielded modest results, with objective response rates in the single-digit range, mostly representing disease stabilization [41]. Slightly better responses have been observed in grade 3 neuroendocrine carcinomas.

For thymic carcinoids specifically, no prospective clinical trials have been conducted to date. Nonetheless, isolated case reports have described encouraging responses to immunotherapy in selected patients [42]. At present, the use of immunotherapy in thymic carcinoids should only be considered in clinical trials [37].

## 8. Prognosis

Thymic carcinoids are associated with a worse prognosis compared to other well-differentiated NETs of the gastrointestinal tract or lung [31]. This is primarily due to their aggressive growth pattern, delayed diagnosis, and frequent presence of metastatic disease at the time of detection [31].

Retrospective studies have identified several prognostic factors, including disease stage at diagnosis, the feasibility of achieving complete surgical resection, histological type and grade, and tumor size [6,31]. In a Surveillance, Epidemiology, and End Results (SEER) database analysis of 160 patients, advanced-stage disease was significantly associated with worse overall survival compared to localized disease (*p* = 0.009), as was the absence of surgical treatment (*p* = 0.005) [1]. Larger tumor size (>8 cm), atypical histology versus typical carcinoid, and the presence of mediastinal lymph node metastases (N1/N2) have also been correlated with inferior outcomes [6].

Functionally active tumors—particularly those secreting ectopic ACTH—are considered negative prognostic indicators, possibly because of complications related to hypercortisolism [22].

Due to the rarity of thymic carcinoids, survival outcomes vary across published series. Overall median survival across all stages is approximately 4–6 years [6]. In the SEER analysis, median overall survival for localized disease exceeded 9 years, whereas for metastatic disease it was less than 3 years [1]. The estimated 5-year survival rate for tumors confined to the thymus ranges from 50% to 70%, while in disseminated disease it drops below 30% [6]. Typical carcinoids have a more favorable prognosis, with some series reporting 5-year survival rates exceeding 80% when tumors are diagnosed early and completely resected [6]. Unfortunately, most thymic carcinoids are atypical or poorly differentiated (G3). Over 80% of patients eventually experience local recurrence or distant metastases during the disease course [18,31], and 10-year mortality rates range from 50% to 60% [31].

Given the high risk of recurrence, close post-treatment surveillance is recommended [5]. Follow-up typically involves clinical assessment and imaging—preferably chest CT, and when indicated, PET or Octreoscan—every 3–6 months during the first 2 years, followed by intervals of 6–12 months for up to 10 years [5]. Some experts advocate lifelong follow-up, particularly in patients with atypical carcinoid tumors [6].

## 9. New Research Directions and Experimental Therapies

The diagnosis and treatment of mediastinal carcinoids remain challenging due to the rarity of the disease. Current research trends focus on several key areas:

• Improved molecular and imaging diagnostics:

Efforts are underway to enhance the sensitivity of diagnostic tools for thymic carcinoids. One promising example is the multigene expression assay NETest (panNETest), which analyzes the expression of 51 genes in blood samples. Preliminary data suggest that NETest may offer higher sensitivity than chromogranin A in detecting disease activity, although its prognostic value, specifically in pulmonary and thymic carcinoids, is still under investigation [5].

In imaging, new radiotracers have been introduced. In addition to 68Ga-DOTATATE, 64Cu-DOTATATE has been developed for PET imaging, providing higher resolution somatostatin receptor imaging [5]. Furthermore, PET using fluorodopa (18F-FDOPA)—a dopamine analog taken up by neuroendocrine cells—is being explored to identify NET lesions that are undetectable on standard 18F-FDG PET scans [5]. These novel techniques have the potential to improve early detection of small lesions and enhance treatment monitoring.

• Targeted therapy:

Recent advances have expanded understanding of the genetic landscape of thymic carcinoids. Frequent mutations in the MEN1 gene, as well as in epigenetic regulators such as ATRX and DAXX, have opened avenues for the development of targeted therapies [6]. Cyclin-dependent kinase (CDK) inhibitors are also under investigation, given that well-differentiated NETs often exhibit disruptions in the RB1/p16 pathway [34].

In a comprehensive, targeted sequencing study, Sakane et al. identified recurrent somatic mutations in TP53 (18.5%), KIT (7.4%), and PDGFRA (5.6%) in thymic neuroendocrine tumors, with 18.5% of cases harboring potentially actionable alterations [32]. Mutations in genes involved in key oncogenic pathways—including p53, RTK/RAS, and PI3K—were found to be the most prevalent, underscoring molecular vulnerabilities that may be amenable to targeted therapy. Importantly, RTK pathway alterations were associated with worse overall survival, highlighting their potential role not only as therapeutic targets but also as prognostic markers. Although EGFR mutations were observed only in a minority of cases (3.7%), their presence raises the possibility of employing EGFR-directed therapies in selected patients. These findings support the value of comprehensive genomic profiling in guiding precision oncology approaches in thymic carcinoids.

Existing targeted therapies, including everolimus and sunitinib, continue to be evaluated within broader populations of pulmonary and thymic carcinoidsto confirm their impact on overall survival [34]. Moreover, next-generation somatostatin receptor antagonists, such as pasireotide (an SSR2 antagonist), are being studied for their potential to more effectively inhibit hormone secretion and tumor growth in carcinoids resistant to octreotide [5].

• Immunotherapy and combination strategies:

As previously discussed, immunotherapy has shown limited efficacy as monotherapy in NETs; however, significant efforts are underway to enhance its effectiveness [37]. One promising strategy involves combination therapies, such as the use of pembrolizumab with everolimus or atezolizumab with a somatostatin analog in patients with advanced NETs [5].

Another innovative approach is radioimmunotherapy, which involves coupling an antibody targeting NET cells with a radioactive isotope—similar to PRRT, but directed against immunologic targets other than somatostatin receptors (e.g., glutamic acid decarboxylase antigen present in some NETs) [37]. It should be noted that radioimmunotherapy for NETs remains in early experimental phases and has not yet been specifically validated for thymic carcinoids.

Additionally, research is ongoing into the application of CAR-T cell therapy targeting the DLL3 antigen, which has already been investigated in small cell lung cancer [37].

Given the extreme rarity of thymic carcinoids, continued data collection through registries and multicenter collaborative studies (such as the European ENETS networks) is essential for the development of evidence-based treatment strategies for these rare malignancies [5].

## 10. Conclusions

TNETs are rare tumors characterized by highly aggressive behavior, even in well-differentiated forms such as TC and AC. Due to their rarity, optimal management strategies remain challenging and are often extrapolated from pulmonary neuroendocrine tumor treatment paradigms. Surgical resection remains the cornerstone of potentially curative therapy, although many patients present with advanced, unresectable disease. The role of adjuvant therapy remains unclear, and systemic treatment options—including somatostatin analogs, cytotoxic chemotherapy, and targeted agents—have shown variable efficacy.

Recent advances in imaging, molecular profiling, and characterization of the immune microenvironment offer new opportunities for improving diagnosis, risk stratification, and therapeutic strategies. In particular, identifying specific molecular alterations in TNETs may facilitate the development of personalized treatment approaches.

A multidisciplinary approach and international collaboration are essential to optimize patient outcomes and to advance clinical and translational knowledge in this rare and complex disease.

## Figures and Tables

**Table 1:** Comparison of typical and atypical carcinoids.

Feature	Typical Carcinoid (TC)	Atypical Carcinoid (AC)
WHO Grade (2021)	Well-differentiated NET, Grade 1	Well-differentiated NET, Grade 2
Histological atypia	Minimal or absent	Present (e.g., nuclear pleomorphism, nucleoli)
Mitotic rate	<2 mitoses per 2 mm^2^	2–10 mitoses per 2 mm^2^
Necrosis	Absent	Focal necrosis present
Ki-67 index (approx.)	<3%	3–20% (may overlap with Grade 3 in rare cases)
Clinical behavior	Indolent, slower-growing	More aggressive, faster progression
Prognosis	Generally favorable (5-year OS > 80%)	Intermediate (5-year OS ~50–70%)

NET—neuroendocrine tumor; WHO—World Health Organization; OS—overall survival.

**Table 2:** Summary of treatment for thymic carcinoids.

Tumor Type	Stage	Surgery	RT	CT	Targeted Therapy/SSA	IO
TC	Localized	Radical resection (preferably thymectomy with lymphadenectomy)	Consider if incomplete resection or risk features	Usually not required	Consider SSA if functioning tumor (e.g., carcinoid syndrome)	No data
AC	Localized	Radical resection, preferably extended thymectomy with lymphadenectomy	Often recommended postoperatively	Consider in high-risk disease (e.g., >10 mitoses, Ki-67 > 10%)	SSA if somatostatin receptor-positive	Potentially, especially in PD-L1 positive tumors
TC/AC	Locoregional	If feasible—neoadjuvant chemoradiotherapy → surgery	Yes, mainly in the adjuvant setting	PE or other regimens (e.g., CAPTEM)	SSA, PRRT if SSTR-positive, Everolimus	Considered (no standard of care)
TC/AC	Advanced	Usually not performed	Palliative	CAPTEM, streptozotocin + 5-FU, cisplatin + etoposide	SSA, PRRT (e.g., 177Lu-DOTATATE)	Investigational, e.g., nivolumab/ipilimumab (for AC)

5-FU—5-fluorouracil; AC—atypical carcinoid; CAPTEM—capecitabine + temozolomide; CT—chemotherapy; IO—immunotherapy; PD-L1—programmed death ligand-1; PE—cisplatin + etoposide; PRRT—peptide receptor radionuclide therapy; RT—radiotherapy; SSA—somatostatin analogs; SSTR—somatostatin receptor; TC—typical carcinoid.
